# Analysis of urinary tobacco-specific nitrosamine 4- (methylnitrosamino)1-(3-pyridyl)-1- butanol (NNAL) and HPV infection in American women: National health and nutrition examination survey

**DOI:** 10.1371/journal.pone.0304499

**Published:** 2024-05-31

**Authors:** Daiwen Liang, Qi Zhang, Wenyu Li, Youkun Lin

**Affiliations:** Department of Dermatology and Venereology, The First Affiliated Hospital of Guangxi Medical University, Nanning, China; University of Connecticut, UNITED STATES

## Abstract

Tobacco-specific nitrosamines (TSNAs) are a group of toxic substances specific to tobacco. 4-(methylnitrosamino)-1-(3-pyridyl)-1-butanol (NNAL) is a tobacco-specific nitrosamine measurable in urine with a much longer half-life than cotinine. We aimed to examine the association between urinary tobacco-specific NNAL and HPV infection among American women. We used cross-sectional data from the National Health and Nutrition Examination Survey (NHANES) between 2007 and 2014 to collect details on their urinary NNAL, HPV infection status, and other essential variables. The association between dietary urinary NNAL and HPV infection status was analyzed by using a weighted multivariate logistic regression model, and stratified subgroup analysis. In total, 5197 participants aged 18–59 years were identified, with overall prevalence of high-risk and low-risk HPV infection of 22.0% and 19.1%, respectively. The highest quartile of NNAL(Q4) was more positively associated with low-risk HPV infection than the lowest quartile of NNAL(Q1) (OR = 1.83 (1.35,2.50), *p*<0.001). the highest quartile of NNAL(Q4) was more positively associated with high-risk HPV infection than the lowest quartile of NNAL(Q1) (OR = 2.20 (1.57,3.08), *p* < 0.001). In subgroup analyses, the positive correlation between urinary NNAL levels and low-risk HPV infection status was inconsistent in marital status and BMI (interaction *p* < 0.05). The positive association of urinary NNAL levels with high-risk HPV infection status was inconsistent in smoking and BMI. (interaction *p* < 0.05). Tobacco-specific NNAL levels positively correlate with high- and low-risk HPV. Future well-designed longitudinal studies are still needed to validate the effect of tobacco exposure on HPV infection by NNAL.

## Introduction

Human papillomavirus (HPV) is one of the most prevalent sexually transmitted diseases in the world, and it is also recognized as the cause of 5% of cancers globally [1]. Most HPV infections are asymptomatic and can be cleared within 12 to 24 months of infection. A small percentage of persistent infections may progress to precancerous lesions. While environmental, host, viral and other factors may affect the various stages of HPV infection, exploring these elements enables us to identify early HPV infection management, prevention, and therapy [2].

In the US, tobacco usage is the main preventable cause of death [3]. There is evidence of a causal relationship between tobacco use and cancers of the lung, larynx, oral cavity, pharynx, esophagus, pancreas, bladder, kidney, cervix, stomach, and acute myeloid leukemia [4]. Meanwhile, several studies have shown that tobacco use and exposure to secondhand smoke are associated with an increased chance of high-risk HPV infection [5–8]. However, some of them have reached the opposite conclusions. HPV16 as the cause of most cervical cancers and one study found no association between its serologic markers and smoking [9]. Therefore, this issue deserves further exploration. Meanwhile most of the available studies used participants’ serum cotinine levels as biomarkers of tobacco exposure. Cotinine is the main metabolite of nicotine, with a half-life of about 16 hours. Tobacco-specific nitrosamines (TSNAs) are a group of toxic substances that, unlike other carcinogens found in cigarettes (e.g., heavy metals), are considered to be specific to tobacco. They are formed in tobacco through the nitrosation of nicotine alkaloids during roasting and fermentation. Among these, nicotine nitrosation produces 4- (methylnitrosamino)1-(3-pyridyl)-1- butanol (NNAL) [10]. Previous studies have found that tobacco-specific NNAL (10–16 days) has a longer half-life compared to cotinine, which is particularly useful when there is a delay between tobacco exposure and the time of biomarker measurement, and therefore may be a better biomarker of tobacco exposure [11]. Currently, there are no studies that use urinary NNAL to detect the association between cigarette exposure and HPV infection in women.

In our study, we utilized data from NHANES to select women aged 18–59 years to study the association between urinary NNAL and HPV infection. The results of this study help to further elucidate the relationship between tobacco exposure and HPV infection.

## Materials and methods

### Data sources and study population

NHANES is a cross-sectional study conducted every two years to assess the health and nutritional status of non-institutionalized individuals in the United States. The NHANES study protocol had NCHS Ethics Review Board approval and all subjects signed a written informed consent form at enrollment. The details of NHANES are available on the official Website. Since this study was a secondary review of data that was made available to the public, it was not subject to ethical declarations, and informed consent was not required. The study included NHANES participants from 2007 to 2014, participants under the age of 18 or over the age of 59 were excluded as the age range for detecting genital infections is 18–59 years. Participants with missing urinary NNAL data, missing HPV data, and missing covariates(age, race, marital status, family income, smoking, sleep hours, first sexual intercourse age, number of sexual intercourses past year, number of sex partners during the lifetime, illegal substance use, drink alcohol consumption, body mass index (BMI), urinary creatinine) were not included in this study.

### Detection and classification of HPV infections

The Linear Array HPV Genotyping Assay (Roche Diagnostic) was used in NHANES to identify 37 HPV genotypes in total (6, 11, 16, 18, 26, 31, 33, 35, 39, 40, 42, 45, 51, 52, 53, 54, 55, 56, 58, 59, 61, 62, 64, 66, 67, 68, 69, 70, 71, 72, 73, 81, 82, 83, 84, 89, and IS39). More details of the testing procedures can be found in the Laboratory Data section of the NHANES website. The outcome variable of the study was the overall status of HPV infection, which was categorized into three groups: negative (negative for all 37 HPV types); high-risk HPV infection positive for at least one of the 14 oncogenic HPV types (16, 18, 31, 33, 35, 39, 45, 51, 52, 56, 58, 59, 66, and 68); and low-risk HPV infection (positive for at least one of the remaining 23 non-oncogenic HPV types and no oncogenic HPV infection) [12].

### Assessment of urine NNAL and covariates

The tobacco-specific nitrosamine NNK (4-(methylnitrosamino)-1-(3-pyridyl)-1-butanone) is a significant component of tobacco and tobacco smoke. In the smoker’s body, NNK is rapidly reduced to its metabolite, NNAL. A significant portion of NNAL may also exist in the glucuronide form NNAL-Gluc (NNAL-N-Gluc and NNAL-O-Gluc) in the urine. Participants’ urine samples are collected during the physical examination and vials are stored under frozen (-30°C) conditions before being shipped to the National Center for Environmental Health for testing. NNAL is measured by using liquid chromatography linked to tandem mass spectrometry (LC/MS/MS). See NHANES website for detailed steps. In cases, where the result was below the limit of detection, the value for that variable is the detection limit divided by the square root of two, reducing bias due to missing data on the left side [13].

According to previous literature [12, 14, 15], our study considered potential confounders such as age, race, marital status, family income, smoking, sleep hours, first sexual intercourse age, number of sexual partners past year, number of sex partners during the lifetime, illegal substance use, drink alcohol consumption, body mass index (BMI), urinary creatinine. Family income was divided into three groups based on the poverty income ratio (PIR): low (PIR ≤ 1.3), medium (1.3 < PIR ≤ 3.5), and high (PIR > 3.5). Smoking status was determined by the question, “Have you smoked at least 100 cigarettes in your entire life”.Alcohol use was determined by the question, “In any 1 year, have you had at least 12 drinks of any type of alcoholic beverage?”. BMI was divided into three groups: normal (BMI<25), overweight (25≤BMI <30), and obese (BMI≥30).

### Statistical analyses

We included weight, class, and PSU variables in all analyses. In the baseline characterization, continuous variables are expressed as weighted means(standard deviations) and categorical variables are expressed as sample numbers(weighted percentages). Participants were categorized into four groups (Q1, Q2, Q3, and Q4) based on quartiles of urinary NNAL levels For continuous variables, differences in baseline characteristics between the four groups were examined using one-way ANOVA (normal distribution) or kruskal-wallis test (skewed distribution), and for categorical variables, chi-square tests were used.

To assess the association between urinary NNAL levels and HPV infection status, we used a weighted logistic regression model, calculating odds ratios (ORs) and 95% confidence intervals (CIs). We made three model sets: The crude model did not adjust for any confounders; Model I adjusted for socio-demographic factors such as age, race, marital status, and household income; and Model II was a fully adjusted model that included adjustments for all factors shown in Table 1. To test for a linear relationship between NNAL and HPV infection status, we used the median of the NNAL quartiles as a continuous variable to calculate *p* for trend.To test for differences in race, marital status, alcohol consumption, smoking, drugs, and BMI between urinary NNAL levels and HPV infection status, we performed subgroup analyses. All statistical analyses were conducted using the EmpowerStats (www.empowerstats.com) and R studio (4.3.1).

**Table 1:** Characteristics of 5197 participants by categories of urinary NNAL.

Characteristic	Urinary NNAL (ng/ml)					*p*-Value
	Total	Q1	Q2	Q3	Q4	
		≤0.0004	0.0005–0.0013	0.0013–0.0323	>0.0323	
No.	5197	2063	572	1263	1299	
Age(years), mean (SD)	39.8 (11.5)	41.9(10.9)	39.0 (11.3)	36.9 (12.1)	39.2 (11.4)	<0.001
Race, n (%)						<0.001
Mexican American	795 (8.3)	424 (9.9)	108 (11.5)	182 (8.7)	81 (3.7)	
Non-Hispanic Black	1111 (12.4)	308 (8.4)	109 (10.8)	363 (17.7)	331 (15.4)	
Non-Hispanic White	2241 (66.8)	824 (67.7)	201 (61.6)	479 (61.6)	737 (72.6)	
Others	1050 (12.4)	507 (14.1)	154 (16.0)	239 (12.0)	150 (8.3)	
Marital status, n (%)						<0.001
Living alone	2148 (36.5)	618 (27.3)	209 (28.6)	634 (44.9)	687 (48.5)	
Living in a couple	3049 (63.5)	1445 (72.7)	363 (71.4)	629 (55.1)	612 (51.5)	
PIR, n (%)						<0.001
Low	1622 (23.7)	407 (11.6)	193 (23.6)	502 (29.2)	701 (40.0)	
Medium	1803 (33.2)	703 (29.3)	197 (34.4)	466 (37.9)	406 (35.1)	
High	1772 (43.2)	953 (59.2)	182 (42.0)	295 (32.9)	192 (24.9)	
Smoke, n (%)						<0.001
No	3204 (59.6)	1713 (80.0)	481 (80.0)	896 (67.3)	114 (6.7)	
Yes	1993 (40.4)	350 (20.0)	91 (20.0)	367 (32.7)	1185 (93.3)	
Sleep hours, n (%)						<0.001
<8	3457 (65.2)	1324 (63.0)	377 (64.7)	831 (65.4)	925 (69.2)	
8–9	1628 (32.9)	710 (35.7)	184 (33.1)	408 (33.1)	326 (27.5)	
>9	112 (1.9)	29 (1.3)	11 (2.3)	24 (1.5)	48 (3.3)	
Age at first sexual intercourse(years), n (%)						<0.001
<16	1367 (24.4)	329 (15.4)	123 (18.4)	336 (26.1)	579 (41.5)	
16–18	2351 (48.5)	868 (45.6)	260 (53.0)	628 (51.2)	595 (48.9)	
>18	1479 (27.2)	866 (39.0)	189 (28.7)	299 (22.6)	125 (9.6)	
No. of sex partners during life, n (%)						<0.001
≤1	863 (15.3)	519 (22.4)	127 (19.4)	172 (12.8)	45 (3.1)	
2–5	2063 (39.2)	866 (40.9)	256 (44.1)	519 (41.3)	422 (32.1)	
6–10	1234 (24.3)	435 (22.8)	125 (23.6)	302 (23.6)	372 (27.8)	
11–50	960 (19.9)	232 (13.5)	62 (12.5)	255 (21.3)	411 (33.5)	
>50	77 (1.3)	11 (0.4)	2 (0.4)	15 (1.0)	49 (3.5)	
No. of sex partners past year, n(%)						<0.001
0	751 (13.6)	327 (14.6)	68 (10.0)	178 (14.5)	178 (12.5)	
1–5	4374 (85.3)	1724 (84.9)	501 (89.1)	1065 (84.3)	1084 (85.1)	
>5	72 (1.2)	12 (0.5)	3 (0.9)	20 (1.2)	37 (2.4)	
Illegal substance use, n(%)						<0.001
No	2559 (43.2)	1302 (53.9)	376 (58.7)	599 (41.6)	282 (18.5)	
Yes	2638 (56.8)	761 (46.1)	196 (41.3)	664 (58.4)	1017 (81.5)	
Alcohol, n (%)						<0.001
No	1569 (24.2)	754 (28.4)	203 (28.5)	368 (23.3)	244 (15.5)	
Yes	3628 (75.8)	1309 (71.6)	369 (71.5)	895 (76.7)	1055 (84.5)	
BMI, n (%)						<0.001
Normal	1688 (35.5)	762 (40.4)	180 (31.6)	333 (27.8)	413 (35.9)	
Overweight	2121 (27.3)	583 (27.7)	160 (29.3)	300 (25.0)	345 (27.7)	
Obese	1388 (37.2)	718 (31.9)	232 (39.0)	630 (47.2)	541 (36.4)	
Urinary creatinine(mg/dL), mean (SD)	106.2 (74.1)	85.3(63.0)	103.4(64.2)	133.67(77.7)	119.15(81.48)	<0.001
HPV infection status, n (%)						
High risk	1261 (22.0)	329 (14.2)	113 (18.9)	388 (28.6)	431 (31.0)	
Low risk	1087 (19.1)	341 (15.1)	105 (17.0)	264 (18.7)	377 (27.7)	
Negative	2849 (58.9)	1393 (70.7)	354 (64.1)	611 (52.7)	491 (41.3)	

## Results

### Basic characteristics

**Fig 1:**
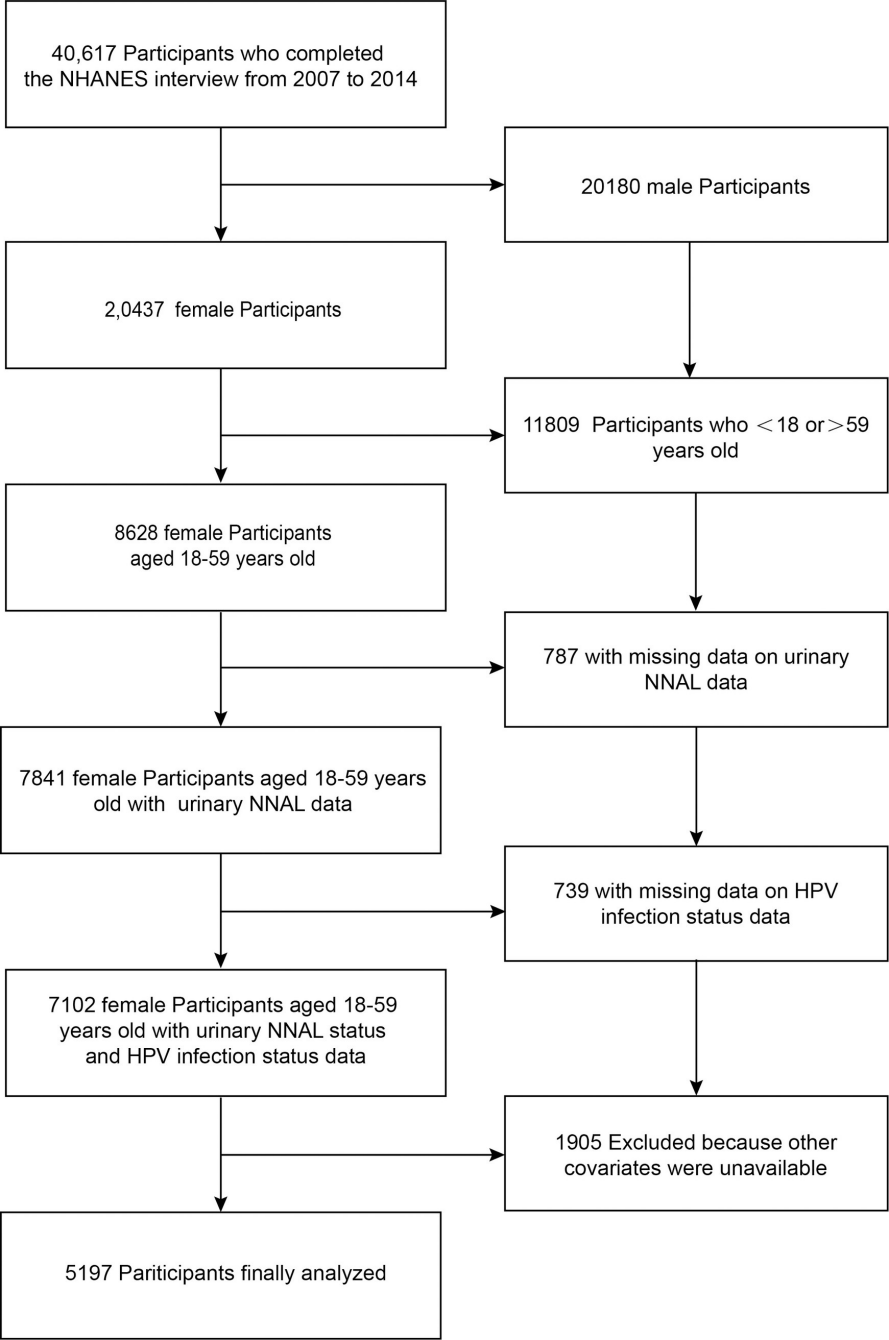
The flow diagram of this study. Abbreviations: NHANES, National Health and Nutrition Examination Survey; HPV, human papillomavirus.

Out of the 40,617 participants in the interview, 20,437 were female. Of these, 11,809 were excluded because they were under 18 or over 59 years old. Additionally, 787 women had missing NNAL data, 739 had missing HPV data, and 1,905 had missing covariates. Ultimately, 5197 women were included in the study. The specific selection process and details are shown in Fig 1. A table of baseline characteristics of individuals grouped by NNAL quartiles is shown in Table 1, The mean age of the 4197 participants in this study was 39.8 (11.5) years, the majority of subjects were Non-Hispanic White(66.8%). Compared to the lowest NNAL quartile(Q1), participants in the highest NNAL quartile(Q4) tended to have low PIR scores, drink alcohol, smoke cigarettes, use illegal substance and be infected the HPV.

### Relationship between urinary NNAL levels and HPV infection

As shown in Table 2, we examined the relationship between urinary NNAL levels and HPV infection status by weighted logistic regression in three models. In the fully adjusted model(Model II), the highest quartile of NNAL(Q4) was more positively associated with low-risk HPV infection than the lowest quartile of NNAL(Q1) (OR = 1.83 (1.35,2.50), *p*<0.001). the highest quartile of NNAL(Q4) was more positively associated with high-risk HPV infection than the lowest quartile of NNAL(Q1) (OR = 2.20 (1.57,3.08), *p* < 0.001). Also by trend test, we found a linear correlation between urinary NNAL levels and HPV infection status.

**Table 2:** Association between urinary NNAL and overall HPV infection.

	Crude ^a^		Model I ^b^		Model II ^c^	
	OR(95%CI)	*P*-Value	OR(95%CI)	*P*-Value	OR(95%CI)	*p*-Value
Low-Risk HPVvs.No HPV						
NNAL(ng/ml)						
Q1	1(Reference)		1(Reference)		1(Reference)	p
Q2	1.24(0.90, 1.70)	0.19	1.17(0.83, 1.65)	0.37	1.13(0.79, 1.63)	0.49
Q3	1.66(1.27, 2.16)	<0.001	1.37(1.04, 1.82)	0.03	1.23(0.93, 1.63)	0.15
Q4	3.14(2.47, 3.98)	<0.001	2.61(1.95, 3.49)	<0.001	1.83(1.35, 2.50)	<0.001
Trend test		<0.001		<0.001		<0.001
High-Risk HPVvs.No HPV						
NNAL(ng/ml)						
Q1	1(Reference)		1(Reference)		1(Reference)	
Q2	1.47(1.07, 2.02)	0.017	1.26(0.90, 1.76)	0.18	1.19(0.84, 1.70)	0.32
Q3	2.70(2.11, 3.46)	<0.001	1.76(1.36, 2.30)	<0.001	1.68(1.26, 2.23)	<0.001
Q4	3.73(2.91, 4.78)	<0.001	2.71(2.00, 3.65)	<0.001	2.20(1.57,3.08)	<0.001
Trend test		<0.001		<0.001		<0.001

Abbreviations: HPV, human papillomavirus; OR, odds ratio; CI, confidence interval. ^a^ No covariates were adjusted in crude model. ^b^ Sociodemographic variables (age, race, marital status, and household income) were adjusted in Model I. ^c^ All covariates presented in Table 1 (age, race, marital status, family income, smoking, sleep hours, first sexual intercourse age, number of sexual intercourses past year, number of sex partners during the lifetime, illegal substance use,drink alcohol consumption, body mass index (BMI), urinary creatinine) were adjusted in Model II

### Stratified analysis based on additional variables

**Fig 2:**
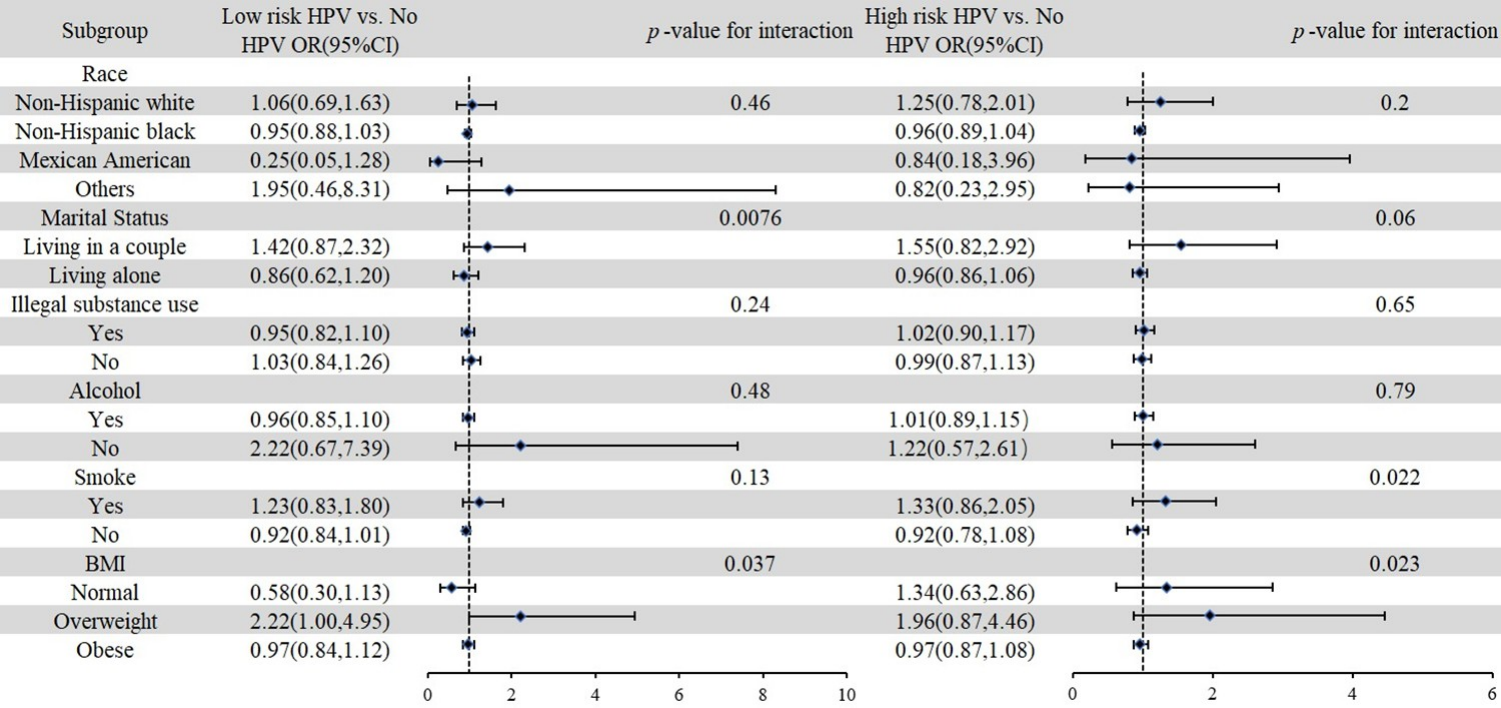
Association between NNAL and HPV infection status in different subgroups. (All covariates presented in Table 1 (age, race, marital status, family income, smoking, sleep hours, first sexual intercourse age, number of sexual intercourses past year, number of sex partners during the lifetime, illegal substance use, drink alcohol consumption, body mass index (BMI)), urinary creatinine were adjusted).

Subgroup analyses and interaction tests were used to test the consistency and stability of the positive association between urinary NNAL levels and HPV infection status. As show in the Fig 2, in the fully adjusted model, the positive correlation between urinary NNAL levels and low-risk HPV infection status was inconsistent in marital status and BMI (interaction *p* < 0.05). The positive association of urinary NNAL levels with high-risk HPV infection status was inconsistent in smoking and BMI (interaction *p* < 0.05).

## Discussion

In this study, we found a significant association between urinary NNAL levels and HPV infection status, which remained significant after adjusting for all covariates. This results suggests that tobacco exposure is associated with HPV infection status.

The current literature on the relationship between tobacco exposure and HPV infection shows a correlation between the two, such as the study by Sadate-Ngatchou P, which found a positive correlation between current smoking and high-risk HPV DNA [6]. Vaccarella S et al. showed that the prevalence of HPV among current smokers increased with smoking intensity [16]. Two cross-sectional studies of serum cotinine that have found an association between tobacco exposure and HPV infection [7, 8]. However, there are also studies that deny this correlation, HPV16 is the cause of most cervical cancers, accounting for about 90% of all virus-positive head and neck cancers [17]. Kelsey KT’s study showing no overall association between HPV16 serologic markers and smoking [9]. However, cotinine has a short half-life, so we chose a longer half-life NNAL for our study. The average half-life of cotinine is 16 hours and the average half-life of NNAL is 10–16 days. Therefore the use of NNAL is expected to be more useful than the use of cotinine in epidemiologic studies with prolonged intermittent exposure to secondhand smoke (SHS) or in studies where biomarker measurements can no longer be made during the time of exposure [11]. In addition to cross-sectional studies, there are also epidemiologic studies and basic mechanistic studies that support our present results.

An ecological time-series modeling study reveals a significant direct association between higher prevalence of smoking and sexually transmitted infections and the incidence of cervical cancer [18]. A follow-up propensity score matching study found that tobacco use may increase the risk of persistent HPV infection, and that this risk is higher for heavier smokers [19]. Tobacco users have higher rates of high-risk HPV infection than non-smokers. Cervical cancer is strongly associated with tobacco use [20]. Epidemiological studies have shown that cigarette smoke carcinogens are cofactors that act synergistically with HPV to increase the risk of cervical cancer progression [21]. A study by Alam et al. found that the presence of the carcinogen benzo[α]pyrene (BaP) in tobacco smoke (TS), and the modulation of the HPV lifecycle by BaP may potentially enhance viral persistence, host tissue carcinogenesis, and tolerance of cancer progression. TS affects both innate immunity and adaptive immune responses [22]. It is now well established that TS is associated with decreased immune system-induced clearance of intracervical HPV and early cervical cancer changes [23, 24]. There is also research supporting the possibility that TS or his specific compounds (e.g., nicotine, BaP, and acrolein) may increase susceptibility to HPV or impair the body’s ability to clear HPV. It was confirmed that tobacco smoke affects HPV replication, while TS may also enhance the oncogenic effects of HPV by promoting DNA damage and thereby enhancing the oncogenic effects of HPV. TS alters gene expression in cells involved in HPV-mediated carcinogenesis [25].

## Limitation

First, NHANES only collected urinary NNAL expression levels from 2007 to 2014, which prevented us from further validating the conclusions of this paper using NHANES data from other cycles. Second, due to the limitation of cross-sectional data, a causal relationship could not be inferred, and longitudinal design studies are still needed to verify this in the future.

## Conclusion

This study reveals an association between urinary NNAL levels and HPV infection status in US women aged 18–59 years, which persists even after accounting for potential confounders.

## References

[1] de Martel C, Ferlay J, Franceschi S, Vignat J, Bray F, Forman D, et al. Global burden of cancers attributable to infections in 2008: a review and synthetic analysis. The Lancet Oncology. 2012;13(6):607–615. doi: 10.1016/S1470-2045(12)70137-7 22575588

[2] de Sanjosé S, Brotons M, Pavón MA. The natural history of human papillomavirus infection. Best practice & research Clinical obstetrics & gynaecology. 2018;47:2–13. doi: 10.1016/j.bpobgyn.2017.08.015 28964706

[3] Balogh E P, Dresler C, Fleury M E, et al.Reducing Tobacco-Related Cancer Incidence and Mortality: Summary of an Institute of Medicine Workshop[J]. Oncologist, 2014, 19(1):21–34. 24304712 10.1634/theoncologist.2013-0230 PMC3903060

[4] Centers for Disease C, Prevention, National Center for Chronic Disease P, Health P, Office on S, Health. Publications and Reports of the Surgeon General. How Tobacco Smoke Causes Disease: The Biology and Behavioral Basis for Smoking-Attributable Disease: A Report of the Surgeon General. Atlanta (GA): Centers for Disease Control and Prevention (US); 2010. 21452462

[5] Tarney CM, Beltran TA, Klaric J, Han JJ. Tobacco Use and Prevalence of Human Papillomavirus in Self-Collected Cervicovaginal Swabs Between 2009 and 2014. Obstetrics and gynecology. 2018;132(1):45–51. doi: 10.1097/AOG.0000000000002681 29889765

[6] Sadate-Ngatchou P, Carter JJ, Hawes SE, Feng Q, Lasof T, Stern JE, et al. Determinants of High-Risk Human Papillomavirus Seroprevalence and DNA Prevalence in Mid-Adult Women. Sexually transmitted diseases. 2016;43(3):192–198. doi: 10.1097/OLQ.0000000000000409 26859807 PMC4748390

[7] Kum-Nji P, Meloy L, Keyser-Marcus L. Tobacco smoke exposure as a risk factor for human papillomavirus infections in women 18–26 years old in the United States. PloS one. 2019;14(10):e0223532. doi: 10.1371/journal.pone.0223532 31665134 PMC6821098

[8] Jiang L, Ma S, Zhang G, Jiang L, Yan L. Analysis of tobacco exposures and high-risk HPV infection in American women: National Health and Nutrition Examination Survey. Environmental science and pollution research international. 2023;30(51):110489–110498. doi: 10.1007/s11356-023-30175-7 37792188 PMC10625505

[9] Kelsey KT, Nelson HH, Kim S, Pawlita M, Langevin SM, Eliot M, et al. Human papillomavirus serology and tobacco smoking in a community control group. BMC infectious diseases. 2015;15:8–15. doi: 10.1186/s12879-014-0737-3 25572638 PMC4296688

[10] Hecht SS. Human urinary carcinogen metabolites: biomarkers for investigating tobacco and cancer. Carcinogenesis. 2002;23(6):907–922. doi: 10.1093/carcin/23.6.907 12082012

[11] Goniewicz ML, Eisner MD, Lazcano-Ponce E, Zielinska-Danch W, Koszowski B, Sobczak A, et al. Comparison of urine cotinine and the tobacco-specific nitrosamine metabolite 4-(methylnitrosamino)-1-(3-pyridyl)-1-butanol (NNAL) and their ratio to discriminate active from passive smoking. Nicotine & tobacco research: official journal of the Society for Research on Nicotine and Tobacco. 2011;13(3):202–208. doi: 10.1093/ntr/ntq237 21330276 PMC3045466

[12] Zhou Q, Fan M, Wang Y, Ma Y, Si H, Dai G. Association between Dietary Vitamin E Intake and Human Papillomavirus Infection among US Adults: A Cross-Sectional Study from National Health and Nutrition Examination Survey. Nutrients. 2023;15(17):3825–3839. doi: 10.3390/nu15173825 37686857 PMC10490162

[13] Cole SR, Chu H, Nie L, Schisterman EF. Estimating the odds ratio when exposure has a limit of detection. International journal of epidemiology. 2009;38(6):1674–1680. doi: 10.1093/ije/dyp269 19667054 PMC2786252

[14] Lin HY, Fu Q, Kao YH, Tseng TS, Reiss K, Cameron JE, et al. Antioxidants Associated With Oncogenic Human Papillomavirus Infection in Women. The Journal of infectious diseases. 2021;224(9):1520–1528. doi: 10.1093/infdis/jiab148 33735375 PMC8599710

[15] Barchitta M, Maugeri A, La Mastra C, Rosa MC, Favara G, Lio RMS, et al. Dietary Antioxidant Intake and Human Papillomavirus Infection: Evidence from a Cross-Sectional Study in Italy. Nutrients. 2020;12(5):1384–1394. doi: 10.3390/nu12051384 32408636 PMC7284420

[16] Vaccarella S, Herrero R, Snijders PJ, Dai M, Thomas JO, Hieu NT, et al. Smoking and human papillomavirus infection: pooled analysis of the International Agency for Research on Cancer HPV Prevalence Surveys. International journal of epidemiology. 2008;37(3):536–546. doi: 10.1093/ije/dyn033 18316350

[17] Gillison ML, Alemany L, Snijders PJ, Chaturvedi A, Steinberg BM, Schwartz S, et al. Human papillomavirus and diseases of the upper airway: head and neck cancer and respiratory papillomatosis. Vaccine. 2012;30 Suppl 5:F34–54. doi: 10.1016/j.vaccine.2012.05.070 23199965

[18] Zheng L, Lin Y, Wu J, Zheng M. The associations of tobacco use, sexually transmitted infections, HPV vaccination, and screening with the global incidence of cervical cancer: an ecological time series modeling study. Epidemiology and health. 2023;45:e2023005. doi: 10.4178/epih.e2023005 36596736 PMC10581889

[19] Ma K, Li S, Wu S, Zhu J, Yang Y. Impact of smoking exposure on human papillomavirus clearance among Chinese women: A follow-up propensity score matching study. Tobacco induced diseases. 2023;21:42–53. doi: 10.18332/tid/161026 36949733 PMC10026377

[20] Utami TW, Kusuma F, Winarto H, Anggraeni TD, Peters AAW, Spaans V, et al. Tobacco use and its association with HPV infection in normal uterine cervix: A study from a Sustainable Development Goals perspective. Tobacco induced diseases. 2021;19:64–71. doi: 10.18332/tid/140093 34413719 PMC8340940

[21] Alam S, Conway MJ, Chen HS, Meyers C. The cigarette smoke carcinogen benzo[a]pyrene enhances human papillomavirus synthesis. Journal of virology. 2008;82(2):1053–1058. doi: 10.1128/JVI.01813-07 17989183 PMC2224590

[22] Qiu F, Liang CL, Liu H, Zeng YQ, Hou S, Huang S, et al. Impacts of cigarette smoking on immune responsiveness: Up and down or upside down? Oncotarget. 2017;8(1):268–284. doi: 10.18632/oncotarget.13613 27902485 PMC5352117

[23] Giuliano AR, Sedjo RL, Roe DJ, Harri R, Baldwi S, Papenfuss MR, et al. Clearance of oncogenic human papillomavirus (HPV) infection: effect of smoking (United States). Cancer causes & control: CCC. 2002;13(9):839–846. doi: 10.1023/a:1020668232219 12462549

[24] Koshiol J, Schroeder J, Jamieson DJ, Marshall SW, Duerr A, Heilig CM, et al. Smoking and time to clearance of human papillomavirus infection in HIV-seropositive and HIV-seronegative women. American journal of epidemiology. 2006;164(2):176–183. doi: 10.1093/aje/kwj165 16775041

[25] Aguayo F, Muñoz JP, Perez-Dominguez F, Carrillo-Beltrán D, Oliva C, Calaf GM, et al. High-Risk Human Papillomavirus and Tobacco Smoke Interactions in Epithelial Carcinogenesis. Cancers. 2020;12(8):2201–2219. doi: 10.3390/cancers12082201 32781676 PMC7465661

